# XEN Glaucoma Implant with Mitomycin C 1-Year Follow-Up: Result and Complications

**DOI:** 10.1155/2017/5457246

**Published:** 2017-03-01

**Authors:** Ahmed Galal, Alper Bilgic, Rasha Eltanamly, Amr Osman

**Affiliations:** ^1^Alpha Vision Augenzentrum, Bremerhaven, Germany; ^2^Research Institute of Ophthalmology, Cairo, Egypt; ^3^Ophthalmology Department, Cairo University, Cairo, Egypt

## Abstract

*Purpose*. To evaluate gel microstent (XEN, Aquesys, Inc) for treatment of primary open angle glaucoma (POAG). *Methods*. In this prospective interventional study, 13 eyes with POAG underwent XEN implantation with subconjunctival mitomycin-C. Of those eyes, 3 were pseudophakic and 10 underwent simultaneous phacoemulsification and XEN. Patients had uncontrolled IOP, had intolerance to therapy, or had maximal therapy but undergoing cataract extraction. Follow-up visits included IOP, number of medications, vision, and complications and lasted for 1 year. Complete success was defined as IOP reduction ≥20% from preoperative baseline at 1 year without any glaucoma medications while partial success as IOP reduction of ≥20% at 1 year with medications. *Results*. IOP dropped from 16 ± 4 mmHg pre-op to 9 ± 5, 11 ± 6, 12 ± 5, 12 ± 4, and 12 ± 3 mmHg at 1 week, 1, 3, 6, and 12 months (*p* = 0.004, 0.026, 0.034, 0.01, and 0.01, Wilcoxon Signed Ranks) consecutively. BCVA (LogMAR) was 0.33 ± 0.34 and improved to 0.13 ± 0.11 at 1 year. Mean number of medications dropped from 1.9 ± 1 preoperatively to 0.3 ± 0.49 (*p* = 0.003) at 1 year. 42% of eyes achieved complete success and 66% qualified success. Complications included choroidal detachment in 2 eyes, and implant extrusion in 1 eye, and 2 eyes underwent trabeculectomy. *Conclusion*. XEN implant is an effective surgical treatment for POAG, with significant reduction in IOP and glaucoma medications at 1 year follow-up.

## 1. Background

Glaucoma is one of the blinding diseases affecting 60 million worldwide; treatment is comprised of medications, lasers, and surgeries [1]. The most common glaucoma surgeries are trabeculectomy and tube shunt drainage devices, lower intraocular pressure (IOP) by diverting aqueous humor (AH) from the anterior chamber (AC) to the subconjunctival space; these surgeries are performed through ab externo approach [2]. Both trabeculectomy and shunt surgery come with a range of complications like hypotony, leakage, shallowing of the anterior chamber, choroidal effusion, and valve-related complications as in encapsulation, tube blockage, erosion, and endothelial cell loss [2]. A novel technique of creating an alternative route through an ab interno approach via implantation of a collagen implant XEN® (AqueSys, CA, USA) has been described in an attempt to overcome the different complications which are seen in both trabeculectomy and shunt operations [3].

XEN implant depends on the Hagen-Poiseuille equation, which allows us to calculate the resistance to flow through a cylindrical tube. Assuming laminar flow of a noncompressible fluid, the outflow resistance and therefore pressure differential increase linearly in relation to the length of the tube and decrease to the fourth power of the lumen radius. A longer thinner tube will provide more resistance to flow than a shorter and wider tube. This equation was used as the principle of XEN implant [4].

This work aims at assessing the results of XEN implants in regard to IOP-lowering effect, number of medications used after surgery, if ever, visual acuity, and possible complications.

## 2. Patients and Methods

This prospective interventional study was conducted in Alpha Vision Augenzentrum, Bremerhaven, Germany. This study included 13 eyes of 10 patients; 6 males and 4 females. All the patients signed an informed consent concerning the procedure, and the study followed the tenants of the Declaration of Helsinki.

The study included 13 eyes with primary open-angle glaucoma (POAG). Glaucoma was previously diagnosed by measured elevated IOP with associated optic nerve head changes detected clinically and confirmed by visual field and HRT.

Three eyes were pseudophakic, and they underwent XEN implantation with subconjunctival mitomycin C. 10 eyes had simultaneous cataract so they underwent phacoemulsification and XEN implantation with subconjunctival mitomycin C.

Inclusion criteria for this study were patients with primary open-angle glaucoma (POAG) with or without cataract already diagnosed and being followed up in our clinic for at least 5 years or those eyes not reaching the target IOP pressure with maximal therapy. Inclusion criteria also included medication intolerance or patients with lack of compliance.

Exclusion criteria included previous trabeculectomy surgery, any possible allergic reaction with the material of the implant, controlled IOP by less than 3 different medications, single-eyed patients, pseudoexfoliation, shallow anterior chamber, and angle closure glaucoma.

Primary open-angle glaucoma was reached in all patients after extensive ocular examination including slit lamp examination, gonioscopy, and IOP measurement by the Goldmann Applanation Tonometry in addition to visual field and HRT.

Ocular examination in the preoperative visit included best corrected visual acuity (BCVA), slit lamp examination, gonioscopy, and IOP measurement by the Goldmann Applanation Tonometry, visual field, and HRT. Postoperative visits were conducted for all patients at 1 day, 1 week, 1 month, 3 months, 6 months, and 12 months after surgery. All patients had to discontinue any prostaglandin topical medications 1 week before surgery.

A minimum follow-up period of 1 year was required from all eyes. In each postoperative visit following examination was performed, visual acuity, IOP measurement, and possible complications were reported. Visual field and HRT tests were performed at 6 and 12 months after surgery. Surgery was performed for all patients under general anesthesia, and the procedure included mitomycin C 0.01% (MMC) subconjunctival injection and insertion of XEN implant with or without cataract extraction.

All prostaglandin analogue medications were discontinued at least for 1 week before surgery.

The XEN implant (AqueSys, CA, USA) is derived from collagen and made of gelatin. The implant is made from cross-linked porcine collagen. The XEN45 implant used in this study has a lumen diameter of 45 microns and a length of 6 mm that would provide aqueous filtration around 2–2.5 mL/min [4]. The soft microimplant provides minimal resistance to flow and is designed to rely on subconjunctival resistance alone. A handheld disposable injector is designed specifically for the surgical implantation of the implant. The inserter has a 27-gauge needle preloaded with the implant.

All surgeries were done by the same surgeon. After skin disinfection, proper field dressing, and speculum insertion, superior nasal conjunctiva was marked 2 and 3 mm from the limbus. Intraoperative 0.1 ml MMC 0.01% was injected subconjunctivally using a 27 G hypodermic needle under tenon and spread with microsponge applied to conjunctiva in the superior nasal quadrant where the implant would be inserted, and it remained for 10 minutes before the implant was injected or in case of cataract extraction before phacoemulsification starts. The MMC was not washed out.

Using an ab interno approach, the preloaded injector needle was inserted through a 1.2 mm corneal paracentesis incision opposite the site of desired implantation after the AC was filled with highly cohesive viscoelastic device. An intraoperative goniolens was used to verify placement through the angle to avoid iris and iris root trauma in all cases. The needle was then directed across the AC and implanted in the target quadrant (usually superonasal). The implant is ideally placed through the scleral spur and tracked 3.0 mm posterior to the limbus exiting through the sclera into the subconjunctival space. Approximately, 2 mm of the implant is left in the AC to provide a connection from the AC to the subconjunctival space which was confirmed by the goniolens. Viscoelastic device was removed from the AC. No further sutures were applied, and at that point, the surgery is terminated.

In cases where cataract extraction was indicated, a main incision was performed at the steepest corneal axis and the paracentesis incisions were performed one nasal and one temporal-inferior at 7 o'clock position and 5 o'clock position for the right and left eyes, respectively. The latest incision was done 2‐3 mm central to the limbus and used for the insertion of the XEN45 into the superior nasal area.

After phacoemulsification was finished, no viscoelastic material was used to implant the intraocular lens (IOL) and was implanted only under BSS being our standard technique in phacoemulsification procedures. After the IOL was properly placed in the bag, the AC was filled with cohesive viscoelastic device and a corneal suture was used to secure the principle 2.4 mm incision. XEN implantation followed as previously indicated. Viscoelastic was promptly removed from AC to prevent XEN implant potential blockage or partial closure after surgery.

Patients were prescribed Isoptomax® (dexamethasone 0.1%, neomycin sulfate, and polymyxin B sulfate, Alcon USA) 4 times a day which was tapered by one drop each week, Predforte® (prednisolone acetate 1%, Allergan USA) twice daily for one month then once for another month, and Ketovision® (Ketolactrometamol Omnivision, Germany) 3 times a day for 3 weeks postoperatively. The follow-up visits were conducted at 1 day, 1 week, and 1, 3, 6, and 12 months postoperatively.

Outcome measured in each visit included BCVA, IOP, medications, possible complications, and management. At 6 and 12 month visits, visual field and HRT tests were done.

Complete success was defined as a postoperative IOP drop of ≥20% from preoperative baseline at 12 months without any glaucoma medications. Partial success was defined as a postoperative IOP reduction of ≥20% at 12 months with medications. Failure was defined as vision loss of light perception or worse, need for additional glaucoma surgery, or <20% reduction of IOP from baseline at 1 year.

In case needling was required, after anesthesia drops were instilled in the eyes together with 2 drops of povidone-iodine 5%; a sterile speculum was inserted. The patient was positioned at the slit lamp, and a sterile 27-gauge insulin syringe was advanced into subconjunctival space adjacent towards the bleb temporally and moved into the subconjunctival space at the same time advancing it toward the scleral flap. The needle was advanced till reaching sclera flap (which was not lifted), and the episcleral adhesions were released. Procedure ended by high bleb formation after aqueous was introduced to the subconjunctival space. No MMC was used during the needling procedure.

### 2.1. Statistical Analysis

Data were statistically described in terms of mean ± standard deviation (±SD) and percentages when appropriate. Comparisons of numerical variables were done using Wilcoxon signed-ranks test, Friedman's two-way analysis test, and Kaplan-Meier survival. All statistical calculations were done using the computer program IBM® SPSS® Statistics 21 (Statistical Package for the Social Science). *p* values less than 0.05 were considered significant.

## 3. Results

Thirteen eyes of ten patients were included in this study. The mean age of the patients was 73.1 ± 10 (58–87) years. All patients had POAG, 10 eyes had XEN implant and cataract extraction on same session, and 3 eyes were pseudophakic.

**Figure 1:**
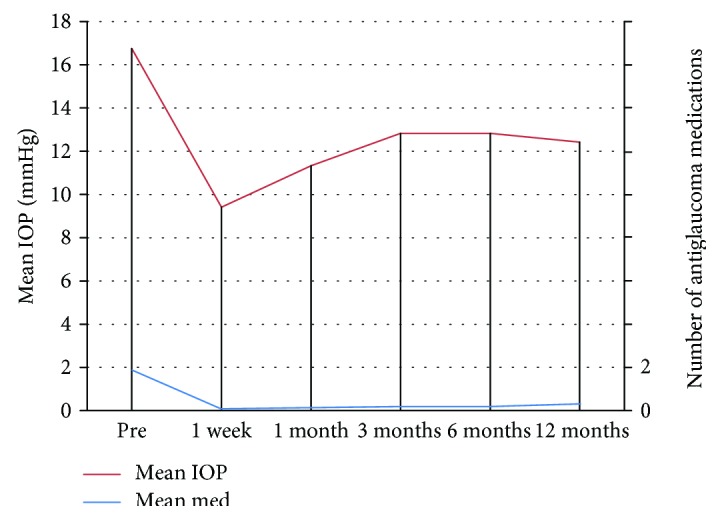
The mean IOP and medication throughout the study.

Mean preoperative IOP was 16 ± 4 (10–24) mmHg which dropped significantly to 9 ± 5 (2–20) mmHg, 11 ± 6 (4–28) mmHg, 12 ± 5 (6–25) mmHg, 12 ± 4 (6–21) mmHg, and 12 ± 3 (6–18) mmHg at 1 week, 1 month, and 3, 6, and 12 months of follow-up (*p* = 0.004, 0.026, 0.034, 0.01, and 0.01—Wilcoxon signed-rank test) consecutively (Figure 1).

There was significant drop of IOP throughout the study (*p* = 0.003—Friedman's two-way analysis). The percent of drop of IOP was 42% at 1 week postoperatively, 30%, 21%, 21%, and 23% at one, 3,6, and 12 months of follow-up.

Mean number of medications dropped from 1.9 ± 1 (1–3) preoperatively to 0.3 ± 0.49 (0‐1) (*p* = 0.003) at 12 months of follow-up.

**Figure 2:**
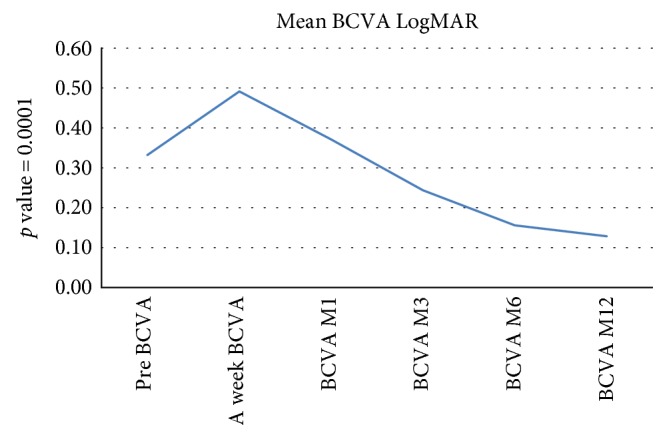
The change of mean BCVA throughout the study.

Preoperative visual acuity was 0.33 LogMAR ± 0.34 (0.0–1.0), which improved significantly to 0.13 ± 0.11 (0.0–0.4) at 12-month follow-up (*p* = 0.0001—Wilcoxon rank test) (Figure 2).

**Figure 3:**
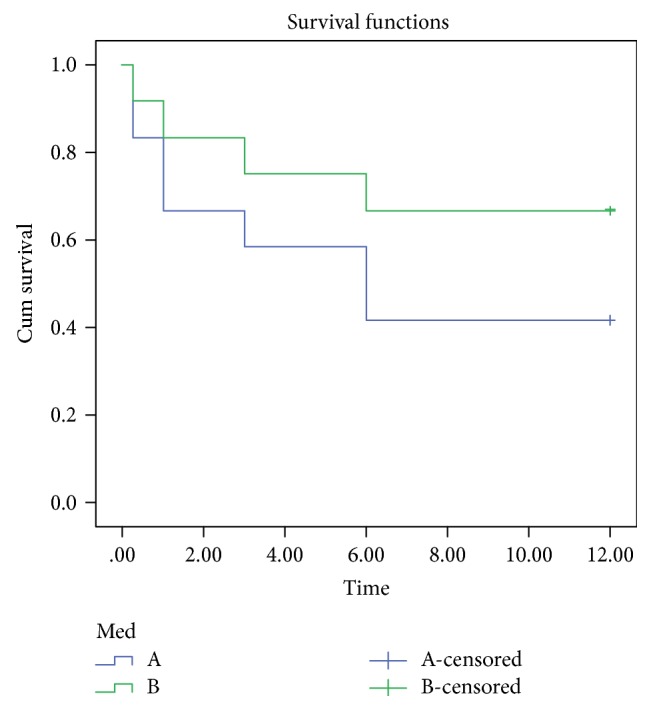
Kaplan-Meier survival curve A complete success and B qualified success.

From the patients included in this study, 41.7% of eyes achieved complete success and 66.7% achieved qualified success by Kaplan-Meier survival curve analysis (Figure 3).

Four eyes (30.7%) required needling during postoperative follow-up, and the needling was done using slit lamp without mitomycin C at 1 month postoperatively in 3 eyes and 3 month postoperatively in the other eye.

Two eyes suffered choroidal detachment and hypotony which were transient and responded to medical treatment in the form of systemic steroids and atropine eye drops. One eye experienced implant extrusion that required repositioning and conjunctival sutures. Two eyes of this study needed further surgical intervention, trabeculectomy, due to inadequately controlled IOP by topical mediations. We did not record severe complications that might affect sight as hyphema, endophthalmitis, choroidal hemorrhage, loss of AC, persistent hypotony, or MMC-related complications.

At 12 months postoperatively, none of the eyes have lost 1 or 2 or more lines of visual acuity. The results of visual fields and HRT at 12 months postoperatively were stable when compared with the preoperative values.

## 4. Discussion

Minimally invasive glaucoma surgery (MIGS) aims to provide a safer, less invasive means of reducing IOP than traditional surgery and to reduce dependency on medications. It is highly debatable whether to include XEN in the MIGS implants although its insertion is done through minimum corneal incision. XEN implant requires mitomycin injection and the formation of a filtrating bleb beside the necessary intraocular manipulation for its insertion; however, this microimplant could not be included as a member of the MIGS. The implant utilizes subconjunctival filtration creating a nonphysiologic route for aqueous outflow which is the basis of the traditional trabeculectomy and aqueous shunt glaucoma surgeries [5].

In this prospective interventional study, we evaluated XEN implant as a new modality shunt device with an ab interno approach sparing the conjunctiva. The implant utilizes the Hagen-Poiseuille equation to achieve the targeted IOP without the presence of a valve.

To our knowledge, there are very few articles in the literature regarding XEN implant in humans.

In this study, there were some social and psychological limitations that prevented us from obtaining the uncontrolled IOP that required the elimination of all the therapeutic lines and left the patients without therapy for several weeks and this somehow was not possible to achieve. All prostaglandin analogue drops were discontinued in all patients for a minimum of 1 week before surgery to decrease the risk of failure and inflammation postoperatively. Surgery was performed for all patients under general anesthesia as indicated by the anesthesia department in our clinic, but we believe it could be done using local anesthesia.

There was a significant drop of IOP from 16 mmHg (±4) to 12 mmHg (±3) at 12 months of follow-up; this was compared to the study by Sheybani et al. [4]. They had preoperative IOP of 23 mmHg (±4.1) and 12 month IOP of 14 mmHg (±3.7). Our mean preoperative IOP was much lower than that of the other study, as some of our cases were initially well controlled on treatment but with intolerance to medications. In our study, 2 eyes had advanced glaucomatous optic nerve damage and they needed lower target IOP.

Preoperative medications decreased significantly from 1.9 ± 1 (1–3) preoperatively to 0.3 ± 0.49 (0‐1) (*p* = 0.003) at 12 months of follow-up, which compare to the decrease from 3.0 at baseline to 1.3 at 12 months (*p* < 0.001) achieved by Sheybani et al. [4].

Our patients achieved a mean IOP reduction of 29.4% at the end of follow-up which was slightly lower than the 36.4% achieved by Sheybani et al.; our cumulative success rate was 41.7% for patients on no medications achieving complete success and 66.7% achieving success on medication, and this again was lower than the partial success rate of 88.9% yet similar to complete success rate of 40.0% achieved by Sheybani et al. [4].

Our procedure differs from that described by Sheybani et al. as we injected 0.01% MMC subconjunctivally without the need to wash it in all our cases; this decreased the rate of needling in our series to 30.7% compared to 47% in their series.

In the tube versus trabeculectomy (TVT) study [6], the mean IOP of the tube arm dropped from 25 ± (5.3) to 12 ± (3.9) at one year of follow-up and dropped from 25 ± (5.3) to 12 ± (5.8) in the trabeculectomy arm. Similar drop was achieved in other studies comparing Ahmed valve to trabeculectomy [7], whereas, in our study, the drop was less from 16 mmHg (±4) to 12 mmHg (±3) at 12 months.

Pérez-Torregrosa et al. performed phacoemulsification combined with XEN45 implant surgery in 30 eyes, and they followed them up to 1 year. Phacoemulsification surgery was performed through 2 temporal incisions, separated by 90°, using the inferior to insert the XEN45 and to implant it in the superior nasal region. The preoperative IOP was 21 ± 3.4 mmHg, with 3 medications and decreased by 29.34% at 12 months. At the end of the study, the number of medications decreased by 94.57%. Complications occurred in 3 eyes, 2 eyes had XEN implantation aborted due to surgical difficulties (subconjunctival hemorrhage and XEN extrusion during preparations), while one eye had filtration bleb failure due to encapsulation 5 months after surgery [8].

Early postoperative complications reported with both tubes and trabeculectomies such as shallow AC, wound leak, aqueous misdirection, suprachoroidal, and vitreous hemorrhage [2, 7] were not encountered in our study yet we encountered two cases of early postoperative hypotony due to choroidal effusion that persisted for less than a month and were treated conservatively using systemic steroids and atropine drops. Late complications such as bleb leak, endophthalmitis, and cystoid macular edema [9] were also not recorded. Complications related to the XEN implant such us exposure of the implant [10] were seen in one case and were managed by applying conjunctival sutures and relocation of the implant in the subconjunctival space. The explanation for this could be that this eye would have had previously scarring and thinning of conjunctiva related to previous nonreported glaucoma procedure. The conjunctival scarring was minimal and was not detected. After implant exposure occurred and was successfully repositioned, the 77-year-old patient recognized that 20 years ago he underwent a nonspecified glaucoma procedure. Although previous failed glaucoma procedure is an exclusion factor for implanting XEN, we did exclude this case to show the potential risk of implanting XEN implant in such a case.

## 5. Conclusion

XEN implant is a recent microshunt device that is implanted through an ab interno approach, thus sparing the conjunctiva for further interference that maybe needed later. This new implant is easy to insert and can achieve reasonable IOP lowering, with minimal complications. Yet, this new technique needs further assessment for longer follow-up survival.
